# Supplementary material for: Associations between Physical Activity and Food Intake among Children and Adolescents: Results of KiGGS Wave 2
Source: Nutrients. 2019 May 11;11(5):1060. doi: 10.3390/nu11051060 (PMC6566319; doi:10.3390/nu11051060)
Supplement: Supplementary file 1 [file nutrients-11-01060-s001.pdf]

Table S1: Threshold values for high intake (top two quintiles) of specific foods in millilitre or gram per day

	High intake					
	Boys			Girls		
	6-10 y	11-13 y	14-17 y	6-10 y	11-13 y	14-17 y
Soft drinks (ml)	≥ 46.4	≥ 121.4	≥ 300.0	≥ 46.4	≥ 85.7	≥ 121.4
Juice (ml)	≥ 203.6	≥ 203.6	≥ 160.7	≥ 203.6	≥ 164.3	≥ 103.6
Water (ml)	≥ 1200	≥ 1200	≥ 1600	≥ 1200	≥ 1600	≥ 2400
Milk (ml)	≥ 300	≥ 300	≥ 300	≥ 300	≥ 200	≥ 200
Dairy products (g)	≥ 122.5	≥ 106.7	≥ 80.7	≥ 113.1	≥ 88.4	≥ 67.5
Fast food (g)	≥ 46.0	≥ 69.6	≥ 100.0	≥ 39.1	≥ 52.5	≥ 60.3
Fruits (g)	≥ 249.1	≥ 152.7	≥ 151.3	≥ 301.3	≥ 208.9	≥ 182.1
Vegetables (g)	≥ 129.2	≥ 97.8	≥ 87.1	≥ 136.6	≥ 115.9	≥ 113.8
Bread (g)	≥ 111.6	≥ 117.9	≥ 133.0	≥ 103.6	≥ 101.8	≥ 100.9
Potatoes/pasta/rice (g)	≥ 124.3	≥ 129.5	≥ 146.0	≥ 119.6	≥ 120.8	≥ 123.7
Meat (g)	≥ 86.5	≥ 109.5	≥ 147.0	≥ 73.3	≥ 86.2	≥ 83.0
Breakfast cereals (g)	≥ 10.1	≥ 11.1	≥ 11.4	≥ 7.3	≥ 7.7	≥ 6.4
Savoury snacks (g)	≥ 3.1	≥ 6.3	≥ 7.6	≥ 2.9	≥ 4.6	≥ 4.3
Confectionery (g)	≥ 76.1	≥ 81.2	≥ 78.3	≥ 70.0	≥ 74.1	≥ 64.4

ml – millilitre; g – gram; y – years
